# The Effects of Overexpressing K2p Channels in Various Tissues on Physiology and Behaviors

**DOI:** 10.3390/insects16080787

**Published:** 2025-07-31

**Authors:** Alaina C. Taul, Elizabeth R. Elliott, Douglas A. Harrison, Robin L. Cooper

**Affiliations:** Department of Biology, University of Kentucky, Lexington, KY 40506, USA; alaina.taul@uky.edu (A.C.T.); dough@uky.edu (D.A.H.)

**Keywords:** behaviors, cardiac, *Drosophila*, K2P, muscle, neurons, potassium channels

## Abstract

Two-pore-domain K^+^ channels (K2p), known previously as leak channels, are responsible for maintaining the resting membrane potential of cells. Fifteen subtypes are known to exist in humans and eleven are known in *Drosophila melanogaster*, as well as six subfamilies; however, little is known about the expression of these subtypes in various animal tissues or the impact of altered expression on cellular physiology. The *Drosophila melanogaster* model allows for selective misexpression of certain neuron subsets, providing insight into individual cell types and the animal’s physiology more generally. Prior research on the overexpression of K2p channels and the resulting behavioral and neuronal effects is limited. This project expanded upon this prior research by using *Drosophila* motor neurons to examine the effects of K2p overexpression on behavior and physiology. After conducting various assays, it was concluded that K2p overexpression in motor neurons had the most prominent effects on *Drosophila* functioning, with sensory, cardiac, and chordotonal neurons also generating differences in behavior. Altered expression levels of K2p channels could result in tissue-specific and/or whole-animal dysfunction.

## Simple Summary

Multiple assays were performed with *Drosophila melanogaster* to investigate the effects of K2p channel overexpression. These assays involved a range of physiological and behavioral characteristics, including climbing (in adults), mouth hook and body wall movement rates (larvae), HAT touch response (larvae), cardiac rates (larvae), membrane electrophysiology (larvae), and light response (larvae). ORK1 K2p channels were chosen as the channel subtype to be overexpressed; additionally, temperature alterations were used to survey how each organism responded to stress, given that overexpression. Behavioral changes were observed even without temperature stress, though such alterations led to some amplification of the effects. The GAL4-induced overexpression of ORK1 in motor, sensory, cardiac, and chordotonal neurons showed differences in membrane potential, body wall movements and heart rates compared to separate driver and responder transgene controls. The m6-m7>ORK1 line had the most dramatic effects across the behavioral and physiological assays (manifesting as more rapid mouth hook movements and less membrane hyperpolarization and light sensitivity). Studying the tissue-specific effects of altered channel overexpression may develop further understanding of how physiological alterations can affect individuals.

## 1. Introduction

Invertebrates—particularly insects—have long represented good model organisms for the exploration of basic physiological concepts and, more indirectly, of human pathological conditions. This stems largely from the fact that such models allow for controlled inducement of genetic/physiological abnormalities and subsequent observation of their effects. In the past, research into common physiological concepts (e.g., sleep, aging, infection, etc.) has been performed using various insect species as models [1,2,3], and the locust served as a primer model in research pertaining to glutamatergic synaptic transmission [4]. Indeed, with the development of new techniques for addressing similarities and differences among species, more interest in unconventional models like these may give rise to comparative studies [5,6].

However, the *Drosophila* model now stands at the forefront of research due to the ease and rapidity of its genetic manipulability and the sheer number of researchers utilizing it [7,8]. *Drosophila* have been used to investigate human pathologies across the gamut: rare diseases, cancer, aging, infectious diseases, and neurological disorders like epilepsy, among others [9,10,11,12]. In the experiments reported herein, the *Drosophila melanogaster* model was applied to several studies into how the impacts of altered ion channel expression on cellular physiology and animal behavior could be addressed.

Cell membrane potential is kept in balance by channels, pumps, and exchangers, some of which—termed leak channels—allow for passive ion movement. Of note are those channels which allow for opposing fluxes of K^+^ and Na^+^ ions. These ions are known to counteract each other and, thus, to form an electrical driving gradient. This gradient may be generally estimated by the Nernst equation for a given permeable ion; however, a better estimation would involve the Goldman–Hodgkin–Katz (GHK) equation, which considers the concentration gradients for several ions and accounts for each ion’s permeability. However, the GHK equation still does not account for pumps and exchangers or variable permeability due to environmental factors (e.g., temperature). Depending on leak channel expression levels, the ion permeability of a whole cell may change. Acute temperature changes can also affect single-channel conductance, thus further impacting whole-cell ion permeability.

The two-pore P-domain potassium channel—or K2p channel—represents the primary channel variety involved in constitutive K^+^ leakage. As mentioned above, these channels play a very large role in the maintenance of resting membrane potential and the control of muscle excitability and contraction, which means that misexpression of K2p channels (e.g., TASK-3 channels) is often observed in conjunction with human pathologies like cancer and epilepsy [13]. Currently, fifteen known K2p channel subtypes exist in humans and eleven exist in *Drosophila* [14,15], belonging to six generally agreed-upon subfamilies: TWIK, TREK, TASK, TALK, THIK, and TRESK [16,17,18,19,20,21,22]. The specifics of these channels vary by subtype, but many can be activated by variations in pH, temperature, and voltage, as well as by mechanical stretching, though the exact sensitivities are also exceedingly variable by type [23,24].

The cells of interest in this study (i.e., skeletal and cardiac muscle cells, neurons) generally have a relatively high K^+^ efflux permeability, with the equilibrium potential for K^+^ ions (E_K_) lingering at some very negative values. In larval *Drosophila*, this has been estimated as resting close to −90 mV for larval body wall muscles [25], which substantiates earlier reports on adult *Drosophila* and moth muscles that estimated the E_K_ potential at close to or more negative than −90 mV [26,27]. The precise equilibrium potential for K^+^ is not known for *Drosophila* cardiac muscles or neurons, though it is likely also a pronounced negative value. *Drosophila* strains genetically modified to bear an overexpression of ORK1 K2p channels in the muscle were found to have a more negative resting membrane potential (RMP): −80 mV, rather than the parental RMP of −55 to −60 mV [25].

Temperature, too, has a significant effect on membrane electrical potential, primarily by altering the driving ion gradient and/or the function of certain K2p channel subtypes [17,21]. As temperature rises, the E_K_ value becomes more negative and, with it, the membrane potential can follow suit, depending on the flux of other ions in the environment. Acute temperature changes may necessitate homeostatic regulation to compensate for the resulting physiological alterations, as the membrane potential varies over time. Temperature changes may also lead to the opening or closing of certain ion channels, such as transient receptor potential channels (TRPs). The temperature-sensitive TRPA1 channels were first described in *Drosophila* but have also been identified and studied in mammals [28]. Responses to environmental changes and sensory stimuli drive organism survival. Reviews of acute, seasonal, and adaptive compensatory mechanisms have been performed in insects, crustaceans, and mammals [29,30,31]; however, this study focuses only on the alterations in behavior and physiology brought about by acute temperature changes.

Presently, very little information is available about K2p channel expression levels in any given cell, or—since K2p channels are modulated by temperature, mechanical stress, pH, membrane potential, and other agents in the environment—the function of various subtypes under different conditions [23,24]. One approach to examining the functions of a given channel subtype would be to knock out (or, alternatively, overexpress) that channel with an RNAi technique and investigate its effects on cellular function, tissue functions, and the body overall. This tactic was chosen for the study reported herein on a large scale as a way of securing a preliminary understanding of how altered K2p channel expression affects a wide array of tissues, thus shedding greater light on physiology and pathological states overall.

Overexpression of dORK1 channels in all muscle fibers is known to result in reduced larval survival [32], while expression in a select subset of muscle fibers allows the larvae to survive into adulthood and reproduce. These ORK1 channels were chosen for this study because this particular channel subtype is known to alter membrane potential when overexpressed and respond to certain pharmacological treatment: properties that bear further investigation [25,33,34]. Additionally, dORK1 channels have a UAS strain, which can be utilized to produce selective expression in various tissues via standard GAL4-UAS genetic crosses. These larvae also showed a more negative membrane potential in the altered muscle fibers compared to controls [25]. All larval *Drosophila* motor neurons excite the muscle, so one might expect alterations in activation and signal conductance to affect movement (locomotion rate) and sensory response as well, which may be exacerbated with increased temperature.

It might seem counterintuitive for hyperpolarization of the muscle to increase synaptic efficacy; however, the ionic driving gradient, EJP amplitude, and spontaneous quantal response amplitude are all enhanced, thus enhancing synaptic strength in turn [35]. Additionally, some cells have a certain percentage of voltage-gated Na^+^ channels kept inactive until hyperpolarization removes that state, thus lowering the activation threshold [35,36]. Given that resting membrane potential can be influenced by the relative expression of K2p (K^+^ leak) channels and NALCN (Na^+^ leak) channels, slight changes in their expression levels can also alter the threshold of voltage-gated channels. It should be noted, however, that, over the course of development, it is possible for compensatory mechanisms to reverse the effects of altered expression and result in larvae with normal behaviors; thus, it was of interest to observe the larvae and their locomotive behavior in an environment that could enhance perturbations produced by altered motor neuron dORK1 expression.

Various larval *Drosophila* sensory neurons respond to mechanical deformation of the cuticle, resulting in a behavioral response [37]. Mechanosensory neurons may also have dual functions, such as the proprioceptive neurons that detect both internal force (exerted on the cuticle by body wall muscle contraction) and external force (the mechanical deformation produced by an external stimulus). Sensory neurons in *Drosophila* primarily use acetylcholine as a transmitter, so targeting the enzyme choline acetyltransferase (CHA) for the co-expression of dORK1 should theoretically ensure that all sensory neurons overexpress the channel. Additionally, class IV and class III dendritic arborization neurons [38]—known also as ppk neurons due to the expression of the pickpocket (*ppk*) gene encoding the degenerin/epithelial sodium channel (DEG/ENaC) family—are mechanosensory neurons involved in nociception responses at the cuticle [38,39] and could also be examined for the effects of dORK1 overexpression. Finally, the effects of overexpression under altered environmental conditions (i.e., increased temperature) also served as an index for the effects of dORK1 overexpression in these neurons. Alterations to sensory information intended for integration within the central nervous system and communication to motor neurons may affect how the motor neurons respond to activity via membrane potential.

In healthy tissues, subtype expression and density vary among cells, even within a given tissue. K2p channel expression is known to be altered under certain pathological conditions [40,41,42], as with the misexpression of TASK-3 channels observed in relation to cancerous tissues [43] and some forms of epilepsy [44]; thus, understanding the effects of ion channel misexpression in model preparations may allow for a better understanding of how such altered expressions affect the cells and the whole animal alike to be developed. In this study, we have used the common GAL4-UAS system to overexpress the *Drosophila* ORK1 gene sequence (i.e., dORK1) in selective cells: motor neurons, sensory neurons, skeletal muscle, and cardiac muscle. However, it should be noted that the precise level of channel overexpression in these specific cell types was not quantified in this study due to limitations in using RT-PCR or RNA sequencing.

## 2. Materials and Methods

### 2.1. Drosophila Lines

**Figure 1:**
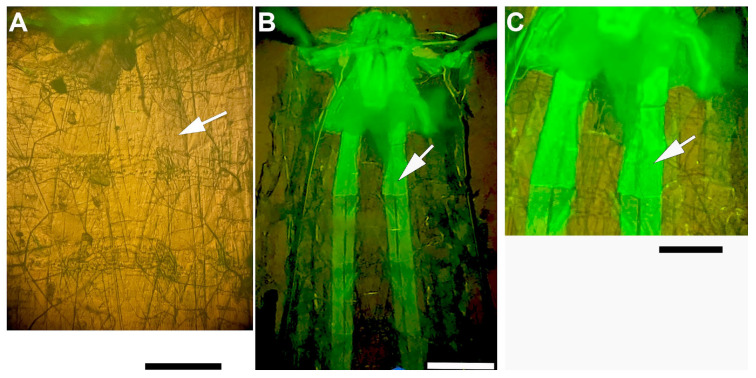
The 3rd-instar larval preparation with co-expression of GFP in m6 and m7 muscles. (**A**,**B**) Dorsal view of the dissected preparation with internal organs removed, showing the ventral body wall muscles and the brain at the top of the figure. (**A**) A UAS-ORK1 larva without GFP expression. Mouth hooks at the rostral end have some auto fluorescence. (**B**) Low magnification was used to illustrate the rostral-caudal nature of GFP-ORK co-expression within m6 and m7 for this strain. (**C**) An enlarged view of the most anterior region of the larvae to illustrate the m6 and m7 muscle fibers expressing GFP. The white arrows indicate m6 muscle fibers. Scale bars: (**A**,**B**) 650 µm; (**C**) 999 µm.

*Drosophila melanogaster* stocks were kept in standard cornmeal fly food medium at 23 °C and 75% humidity on a 12 h light/dark cycle. The following strains were used: Canton S (wild type); D42-GAL4 (all motor neurons; BDSC; catalog no. 8816), ppk-GAL4 (class IV and class III dendritic arborization neurons; BDSC; catalog no. 32078), iav-GAL4 (chordotonal neurons in the pattern of the *iav* gene [45,46] BDSC stock # 36360 and 52273), BG487-GAL4 (larval body wall muscles m6 and m7 in an anteroposterior gradient in larval body wall muscles 6/7 (Figure 1); BDSC stock # 51634) [47,48,49]; CHA-GAL4: UAS-GFP (sensory neurons using choline acetyltransferase to make acetylcholine BDSC stock # 6793). The UAS line used was: UAS-ORK1 (BDSC stock # 6586). A heart-specific strain, Hand4.2-Gal4 (on II; gift from Dr. Anthony Cammarato), was used for expression of ORK1 in cardiac tissue. Control animals for GAL4 and UAS lines used for behavior and physiology assays were generated by crossing the driver or UAS responder lines alone to a *y^1^ w^1^* strain (gift of N. Perrimon). The outcrossed heterozygous transgene animals could then be compared across a common outcross background. In the text, GAL4/UAS transgenes combined in crosses are designated as GAL4>UAS. Control outcrosses of individual transgenes are simply designated as “XXX-GAL4” or “UAS-ORK1”.

### 2.2. Behavioral Assay: Climbing

The climbing assay was the only behavioral assay in this study performed with adult *Drosophila*. Males were selected 2 to 3 days post-eclosion and transported to individual plastic tubes (produced by taping together two *Drosophila* culture cylindrical vials of dimension 1−1/4′′ diameter × 4′′ tall; https://www.geneseesci.com/product/flystuff-drosophila-vials/ (accessed on 28 July 2025).), whereupon they were left for one minute to recover from the CO_2_ exposure. The sealed tubes were tapped against a tabletop to knock the flies to the bottom of the tube, after which they were allowed to climb up the sides of the vessel. The time it took the flies to cross the midline (4 inches; 10.16 cm) was measured. Using unique tubes removed any potential chemical cues that might come from reusing the same vessels.

### 2.3. Behavioral Assay: Mouth Hook and Body Wall Movements

Early third instar larvae were used for behavioral assays. Locomotory and feeding behaviors were assessed as described in Neckameyer [50] and Li and Cooper [51]. In brief, single animals were placed on a filter paper moistened by apple juice and the number of body wall contractions counted for one minute. Immediately afterwards, the animal was placed in a Petri dish containing 2% Baker’s yeast solution to the point of just covering the larva while nonetheless allowing the spiracles to reach out of the solution. In this condition, *Drosophila* larvae immediately feed, initiating a pattern of repetitive mouth hook movements. The number of full mouth hook contractions in one minute was counted [52]. The results of these behaviors were plotted as body wall contractions or mouth hook movements per minute.

### 2.4. Behavioral Assay: Head Abdomen Tail (HAT) Assay

This assay for a behavioral response to touch was followed as previously described [37]. In brief, the dorsal aspect of the larvae was touched with a fine monofilament fiber in three locations: (1) at the most rostral region, over the mouth hooks, (2) about mid-body (i.e., the abdomen), and (3) at the most caudal region. Generally, we refer to this as a HAT assay (i.e., head, abdomen, tail), though the order in which these locations were tapped was randomized from trial to trial. For example, one preparation could receive stimulus first at the head, then the abdomen, then the tail, while another might have received stimulus at the abdomen first, then the head, then the tail, and so forth. This was intended to reduce confounding, as behavioral responses to a stimulus might be affected by the stimuli before/after it. Behavioral responses were matched to a previously developed ethogram (see Appendix A). The range of applied pressures has previously been determined to be 4.5–5.5 g (44.1–53.9 mN) [35].

### 2.5. Physiological Assay: Cardiac Rate

Cardiac measurements were taken from early 3rd-instar larvae. Prior to measurement, dissection to expose the larval heart tube was performed via a ventral incision and pinning of the four corners, the details of which have previously been shown in video format [52,53]. Guts and all visceral organs were removed in such a way as to leave the heart intact and still attached to both ends of the larvae. This dissection technique takes 3–6 min and has been used to directly assess the effects of pharmacological agent exposure on the heart of *Drosophila* larvae [53,54]. Each preparation was allowed to relax for 3–5 min after dissection while bathed in HL3 saline. The movements of the in situ heart were observed with a dissection microscope (adjustable zoom 0.67–4.5; World Precision Instrument, FL, USA, fitted with a 10· eye objective) and the heart rate directly counted. The physiological saline was composed of hemolymph-like fly saline (HL3): (in mmol/L) 70 NaCl, 5 KCl, 20 MgCl_2_, 10 NaHCO_3_, 1 CaCl_2_, 5 trehalose, 115 sucrose, 25 N,N-bis(2-hydroxyethyl)-2-aminoethane sulfonic acid (BES), and pH was kept at 7.1.

### 2.6. Video of Response to White Light

To compare the effects of white light on early 3rd-instar larvae of the M6-M7>ORK1 and UAS-ORK strains, they were dark-adapted for 10 min. Video recordings were taken with an IR camera while they crawled. Once the larvae were all observed to be crawling, they were then exposed to a dim white light. This was repeated with different groups of larvae, including a UAS parental group and an overexpressing group of M6-M7>ORK1 for easy observation of the differences in white light response. The camcorder used was a Panasonic 4K video Camera Model HA-VX870 (2nd Floor, Two Riverfront Plaza, Newark, NJ, USA 07102-5490) recorded at 72 Mb per second for 3840 × 2160 resolution and was able to rapidly adjust to alterations in lighting.

### 2.7. Membrane Potential with Temperature

Temperature, like other external factors, is known to influence cell membrane potential; thus, the potential of larval muscles overexpressing ORK1 channels was tested. The transmembrane potential of a muscle fiber is relatively easy to monitor in this model and is performed using sharp microelectrodes. The resting membrane potential was measured before and during acute temperature changes in M6-M7>ORK1 strain from early-third-instar larvae dissected as previously described [25]. A modified basal HL3 saline at pH 7.2 was used. Muscle m6 in segment 2 was impaled with a sharp intracellular electrode (30 to 40 megaohm resistance) filled with 3M KCl, and an Axoclamp 2B (Molecular Devices, Sunnyvale, CA, USA) amplifier and 1 X LU head stage were used to record the data. Preparations were monitored at 21 °C, after which the temperature was acutely increased to 33 °C via bathing medium exchange, then allowed to drift back to 21 °C.

### 2.8. Developmental Studies

To address the effects of altered channel expression on growth and development, the time from 1st instar larva to pupation was measured for each GAL4/UAS-ORK1 cross and for each individual transgene line outcrossed with a control *y^1^ w^1^* strain. Ten plastic vials, each with ten 1st-instar larvae were used for assessment. All larvae were placed on the food and cotton lids used to create an aerated and moist environment (21 °C). The larvae were then left to develop into pupae and the number of pupae per vial was counted each day. In this manner, the time necessary for pupation was measured for each strain.

## 3. Results

Behavioral studies were conducted in *Drosophila* with and without ORK1 overexpression in different tissues and cell types, and the results can be generally summarized as follows: ORK1 overexpression often resulted in different behaviors than those observed in the parental transgenic lines alone even without the additional stressor of higher temperatures, while exposure of experimental larvae to heat saw accentuation of some of these effects. Additionally, larval behavioral assays revealed more prominent differences than those observed via an adult assay.

### 3.1. Behavioral Assay: Climbing

**Figure 2:**
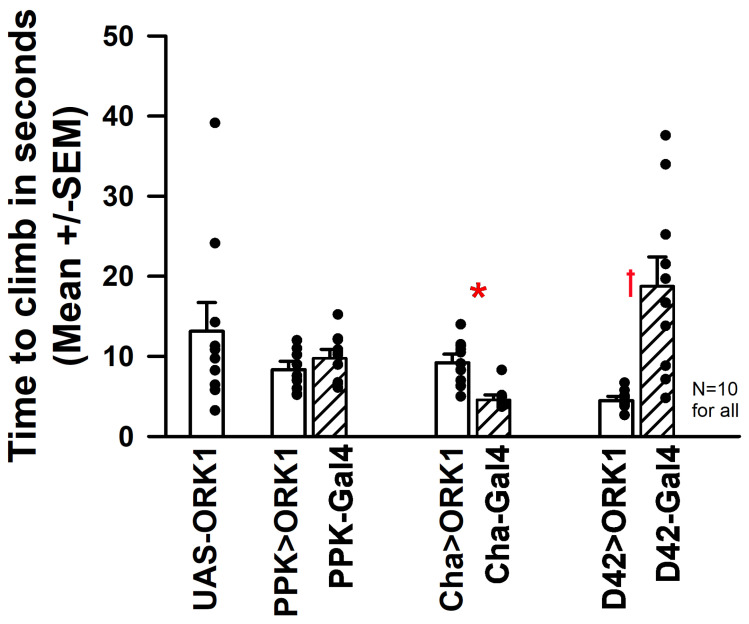
The adult climbing assay for sensory and motor neurons overexpressing ORK1 and controls. Significant differences were observed for Cha-Gal4 and Cha>ORK1 (Figure 2: * *T*-test *p* < 0.05) as well as D42-Gal4 and D42>ORK1 (Figure 2: † Mann–Whitney Rank Sum Test *p* < 0.05). When comparing all the Gal4 lines to each other and to ORK1-UAS, significant differences were observed between the ORK1- UAS and Cha-Gal4 lines and the Cha-Gal4 and D42-Gal4 lines (ANOVA, *p* < 0.05). Comparison of UAS-ORK1 to ORK1-overexpressing lines resulted in significant differences with only the D42>ORK1 preparations (ANOVA, *p* < 0.05).

The climbing assay measures the time taken for young adult males to climb a set distance along the wall of a sealed vial from the base of the tube (Figure 2). Each GAL4/UAS-ORK1 combination was compared to UAS-ORK1 and GAL4 drivers individually outcrossed with *y^1^ w^1^*. Significant differences were observed for Cha-Gal4 and Cha>ORK1 (Figure 2: * *T*-test *p* < 0.05) as well as D42-Gal4 and D42>ORK1 (Figure 2: † Mann–Whitney Rank Sum Test *p* < 0.05). In comparing all the Gal4 lines to each other and to ORK1-UAS, it was found that the ORK1- UAS and Cha-Gal4 as well as Cha-Gal4 to D42-Gal4 were significantly different (ANOVA, *p* < 0.05). Comparing UAS-ORK1 to the ORK1-expressing lines, only the D42>ORK1 was significantly different (ANOVA, *p* < 0.05).

### 3.2. Behavioral Assay: Mouth Hook and Body Wall Movements

**Figure 3:**
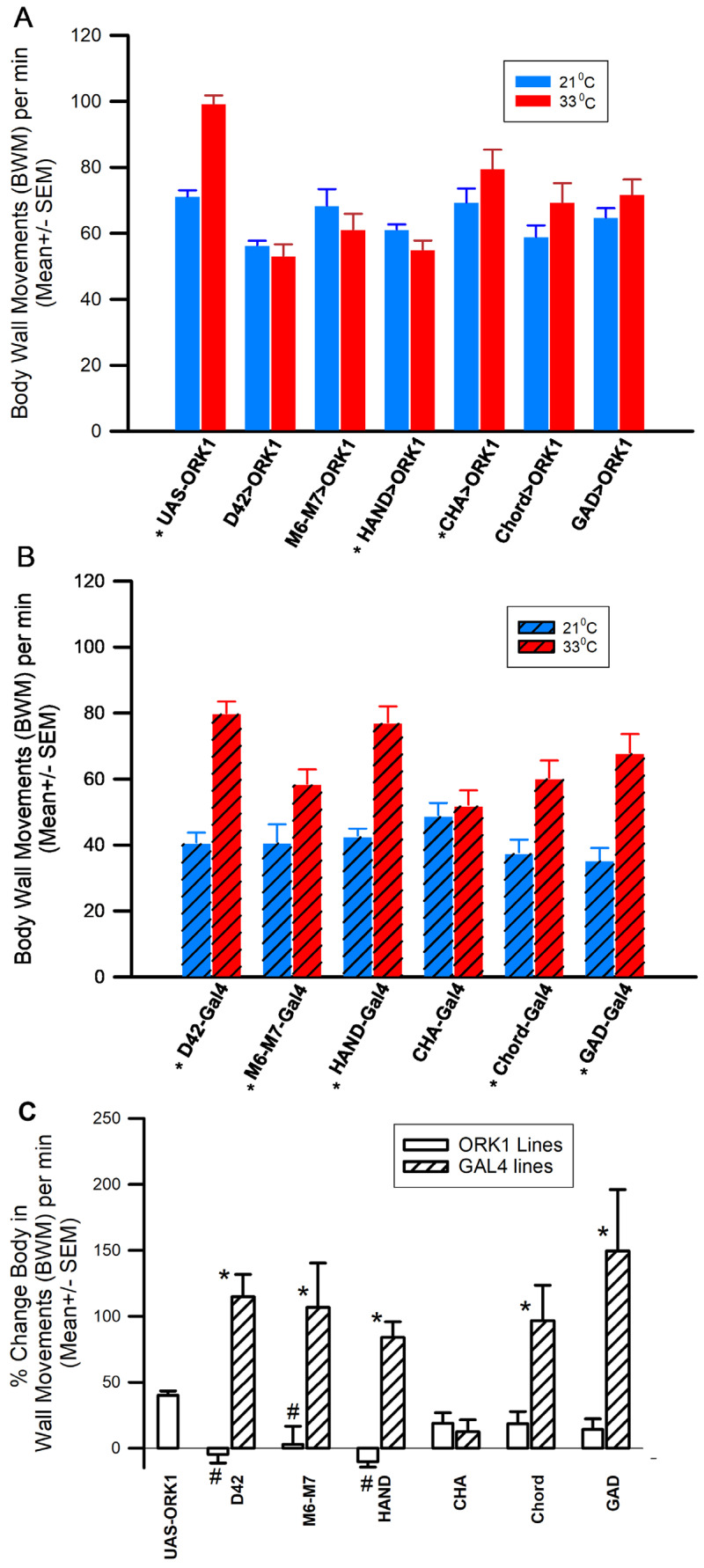
Locomotion is dampened by heat stress and by ORK1 overexpression. (**A**) Overexpression of ORK1 K2p channels led to fewer body wall movements than UAS-ORK1 control at 33 °C but further temperature alteration resulted in no significant responses. (**B**) With the exception of CHA-GAL4, outcrossed GAL4-driver-controlled flies showed an increase in locomotion with increased temperature, similar to UAS-ORK1 alone. Since the parental control group and the crosses with y^1^w exhibited a drastic increase in locomotion during exposure to heat, ORK1 channel overexpression seems to alter the stress response from expected norms. The asterisks represent significant changes within a group (*p* > 0.05, *T*-test, *n* = 20 each group). (**C**) The percent changes in body wall movements (BWMs) from 21 to 33 °C illustrate the differences between the lines with overexpression of ORK1 as compared to the GAL4 lines and the UAS-ORK line. The open boxes are GAL4>ORK1 and the striped boxes are GAL4 only. All of the GAL4 lines were significantly different from all of the UAS lines, with the exception of CHA>ORK1 and Cha>GAL4 (*T*-test, * *p* < 0.05, *n* = 20 for each). Comparison of the percent changes in the UAS-ORK1 compared to the tissue-specific overexpression showed the following were significantly different from one another: D42>ORK1, M6M7>ORK1 and HAND>ORK1 (Figure 3C, ANOVA, # *p* < 0.05).

Larval behaviors were observed to be significantly different in strains overexpressing ORK1 channels, even without additional stressors from a higher temperature environment; however, acute exposure to heat resulted in more pronounced effects on some strains compared to others. The temperature increase resulted in an increased locomotive rate (i.e., body wall movements; BWMs) for all GAL4>ORK1 flies (Figure 3A, *p* > 0.05, *T*-test, *n* = 10 each group), as well as for all individual transgene outcrosses, except CHA-GAL4 (Figure 3B, *p* > 0.05, *T*-test, *n* = 10 each group). The percent changes in body wall movements from 21 to 33 °C illustrate the differences between the lines with overexpression of ORK1 as compared to the GAL4 lines. The percent changes in BWMs from 21 °C to 33 °C presented a compelling result in that the lines with an overexpression of ORK1 resulted in a reduced effect of exposure to an increased temperature compared to the GAL4 lines (Figure 3C). All the GAL4 to UAS lines were significantly different from one another with the exception of CHA>ORK1 and Cha>GAL4 (*T*-test *p* < 0.05, *n* = 20 for each). A comparison between the percent changes in the UAS-ORK1 and the specific tissue overexpressors showed the following significant differences: D42>ORK1, M6M7>ORK1 and HAND>ORK1 (Figure 3C, ANOVA, # *p* < 0.05).

**Figure 4:**
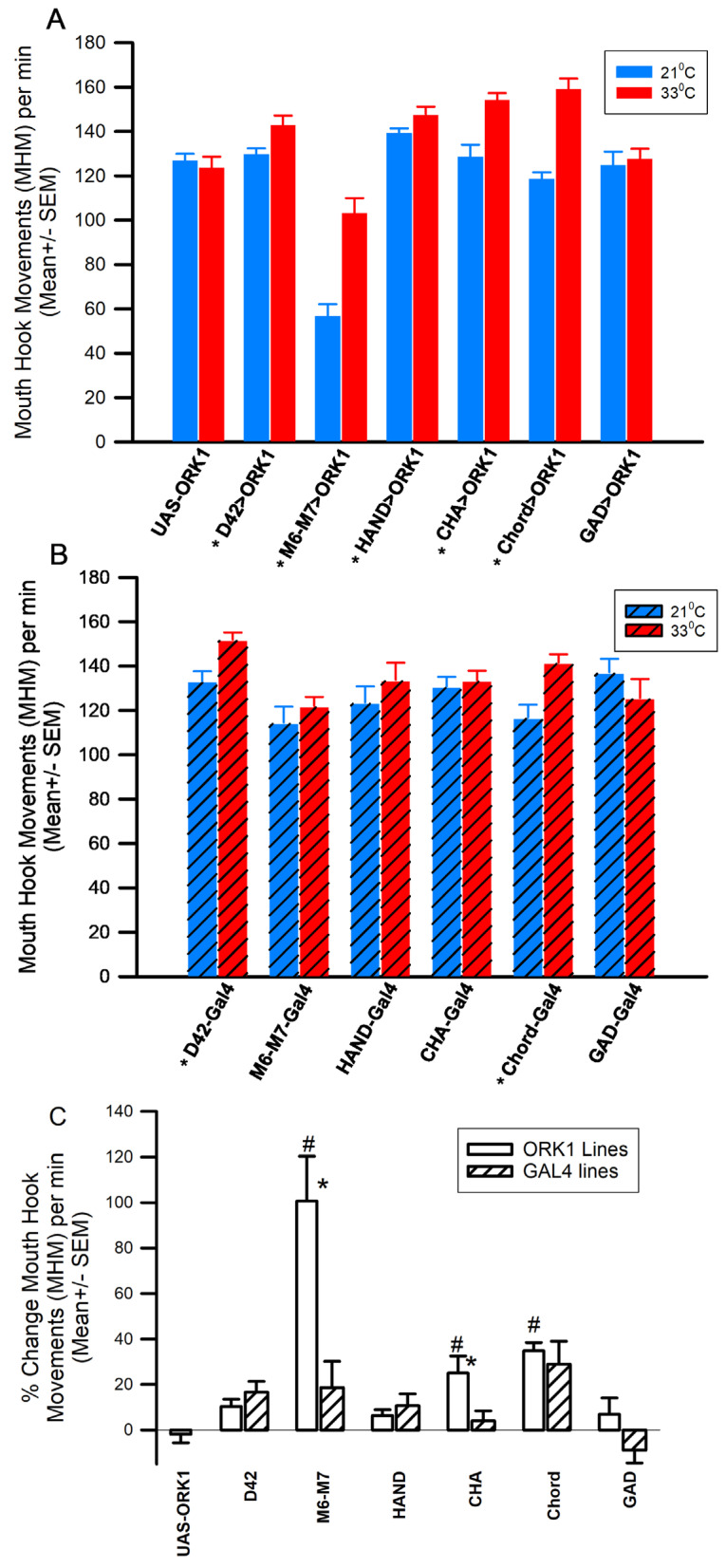
Mouth hook movements. (**A**) All ORK1-overexpressing lines save GAD>ORK1 had a significantly increased feeding rate (i.e., mouth hook movements per minute; MHMs) with increased temperature, while the parental UAS-ORK1 had no significant response to temperature alterations. M6M7>ORK showed a lower rate even at 21 °C and 33 °C compared to other strains overexpressing ORK1 (ANOVA *p* < 0.05). (**B**) For the GAL4-only controls, only D42-GAL4 and Chord-GAL4 showed an increase in mouth hook movements after an increase in temperature (* *p* < 0.05, *T*-test, *n* = 20 for all groups). (**C**) The percent changes in MHMs from 21 to 33 °C illustrate the differences between the lines with overexpression of ORK1 as compared to the GAL4 lines and the UAS-ORK line. The open boxes are GAL4>ORK1 and the striped boxes are GAL4 only. The two groups which showed a significant difference between the ORK1 and GAL4 lines were M6M7 and CHA (*T*-test, * *p* < 0.05, *n* = 20 for each). A comparison of the percent changes in the UAS-ORK1 to the tissue-specific overexpressors showed that the following were significantly different from one another: M6M7>ORK1, CHA>ORK1 and Chord>ORK1 (Figure 3C, ANOVA, # *p* < 0.05).

It is interesting to note that the changes observed in eating rate (as measured via mouth hook movements, MHMs) differ from those changes observed in body wall locomotion (Figure 4). Specifically, the D42>ORK1, M6M7>ORK1, HAND>ORK1, CHA>ORK1, and Cord>ORK1 strains all saw increased eating rates (Figure 4A, * *p* < 0.05, *T*-test, *n* = 20 for all groups), while the parental UAS-ORK1 did not exhibit such an increase. (A comparison of the percent changes between the UAS-ORK1 preparations and the tissue-specific overexpressors revealed significant differences for M6M7>ORK1 and Cha>ORK1. Figure 4C, ANOVA, # *p* < 0.05.) The largest percent change was observed in the M6-M7>ORK1 strain, for which exposure to 34 °C resulted in, on average, a 78% increase in the mouth hook movement rate (Figure 4A). However, for lines outcrossed with *y^1^ w^1^*, only D42-GAL4 and Chord-GAL4 flies showed a significant increase in mouth hook movements during exposure to increased temperatures (Figure 4B, *p* < 0.05, *T*-test, *n* = 20 for all groups). Statistically significant differences from controls in responsiveness to elevated temperature are illustrated by the percent changes in MHMs from 21 °C to 33 °C (Figure 4C) for the M6M7>ORK1- and Cha>ORK1-overexpressing fly larvae (M6M7>ORK1 vs. M6M7-GAL4 and Cha>ORK1 vs. Cha-GAL4; *T*-test, * *p* < 0.05; Figure 4C). These results suggest that both the body wall muscles and sensory neurons contribute to the temperature hyperactivity in feeding displayed by ORK1-overexpressors.

### 3.3. Behavioral Assay: Head Abdomen Tail (HAT) Assay

**Figure A1:**
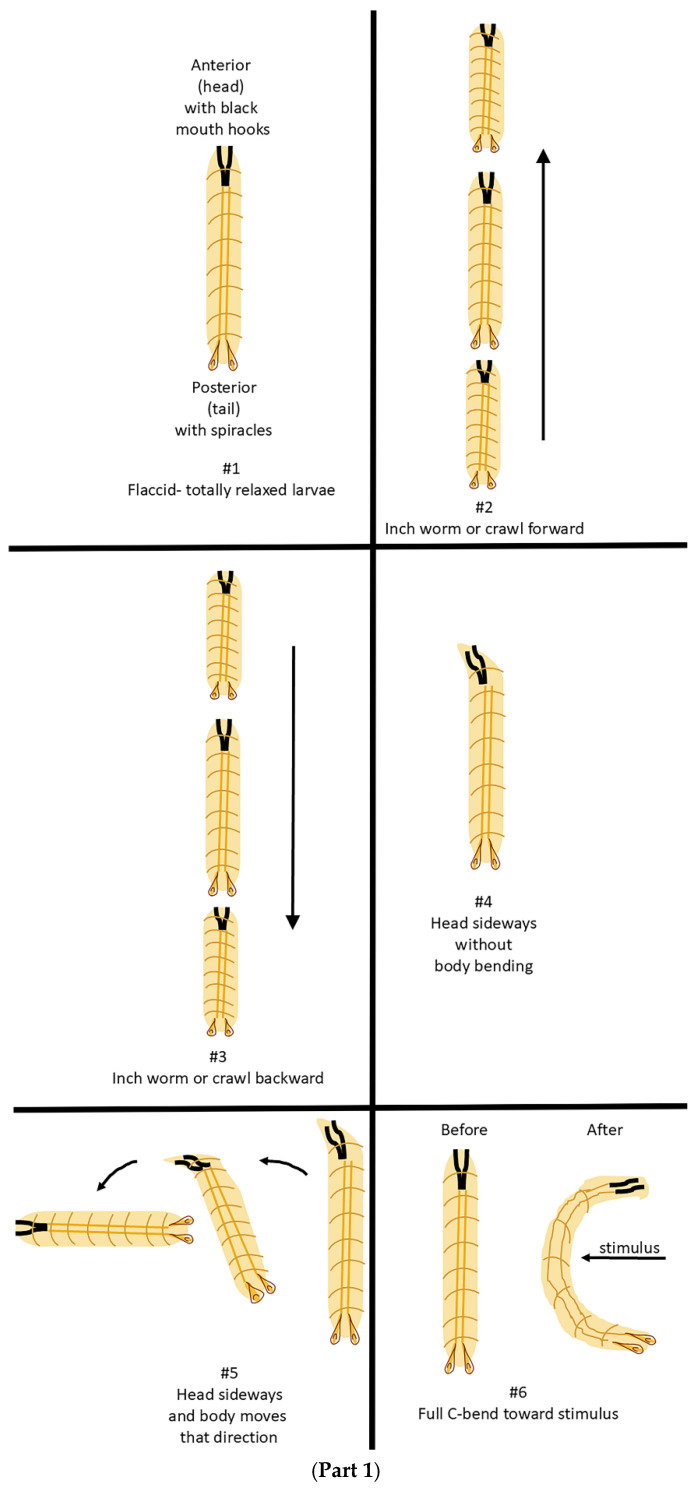

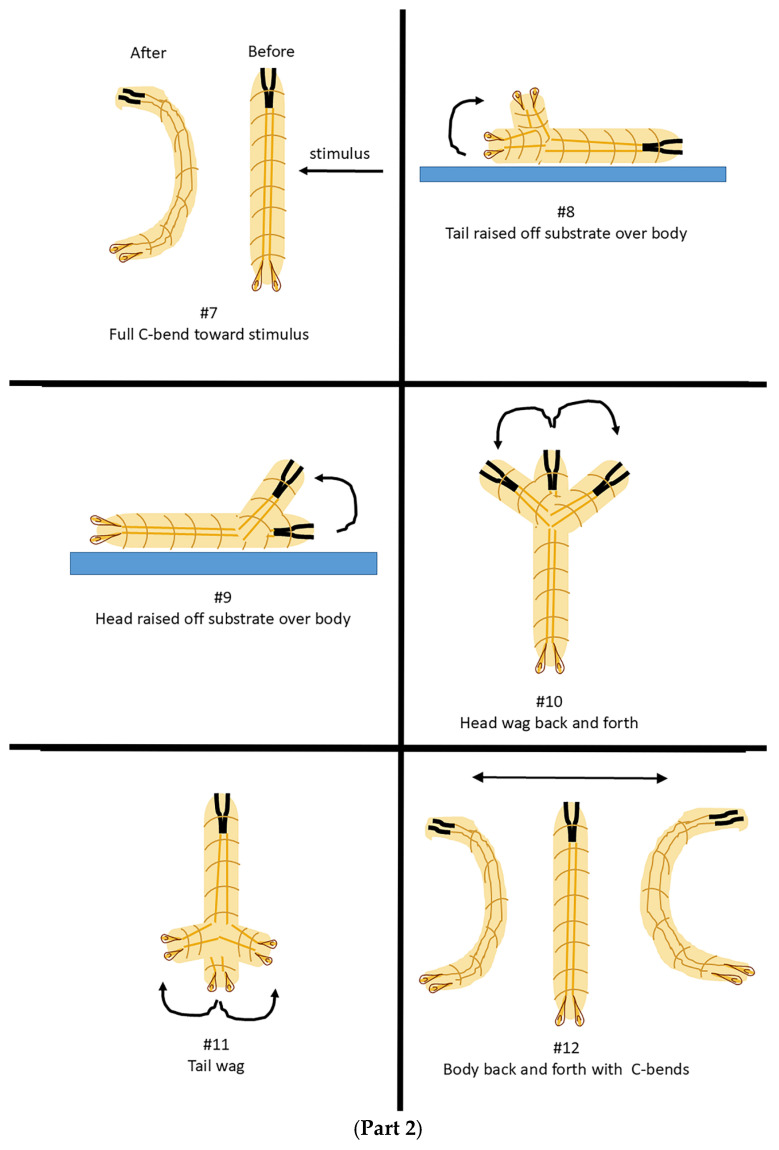

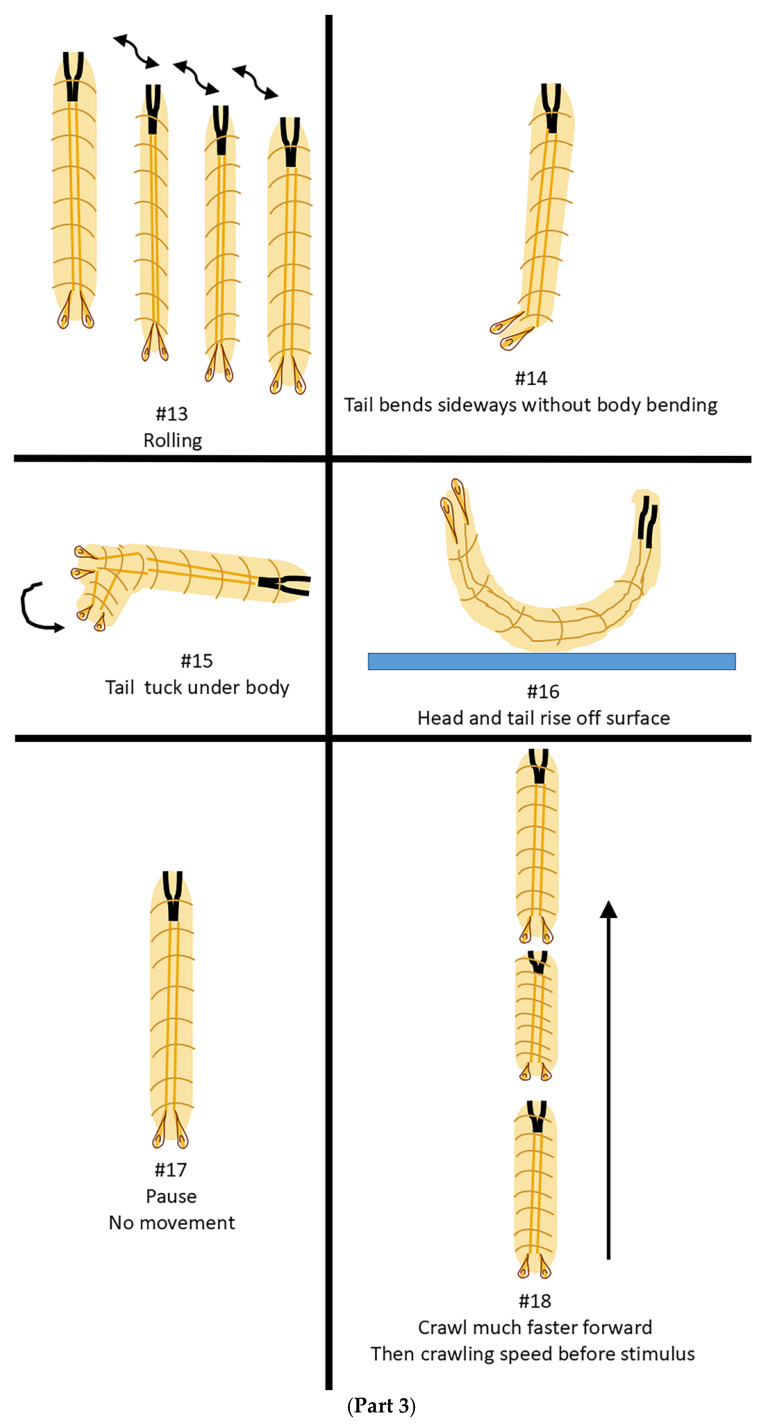
The index of larval behaviors (Part 1); The index of larval behaviors (Part 2); The index of larval behaviors (Part 3).

**Figure A2:**
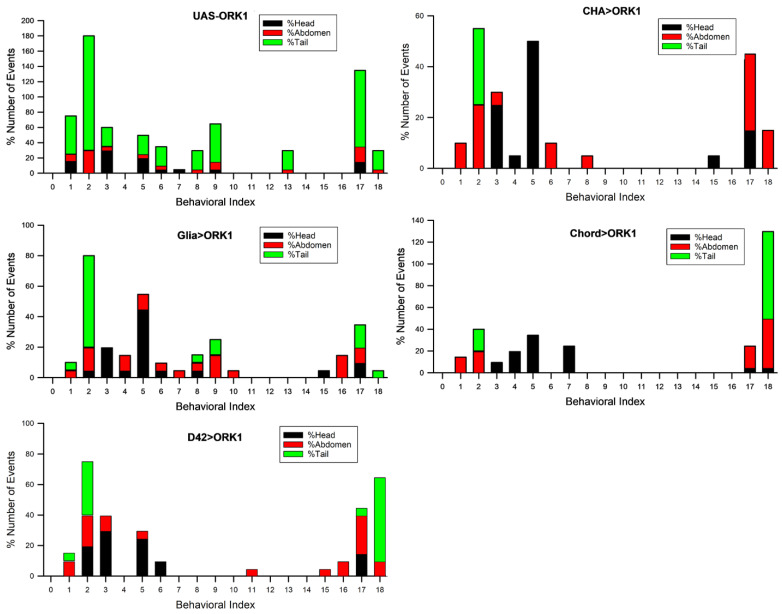
HAT (head, abdomen, tail) behavioral assays with ORK1 channel overexpression in different cell types. The relative percentage of behavioral response occurrences for the twenty trials is displayed for each strain. The ethogram index is presented in the Appendix and describes eighteen possible responses. This assay was performed at 21 °C.

Investigations into *Drosophila* touch sensitivity via the HAT assay suggest that touch response depends on which region of the larvae is being physically stimulated. To start building a sense of whether specific movements are characteristic of particular tissue strains expressing transgenes, such as ORK1, a preliminary study was conducted. Different behavioral responses were observed depending on both the region being stimulated and the strain being investigated. The types of responses observed following contact to the heads, abdomens, and tails of the larvae are indicated below using the ethogram index used for testing (Figure A1). The responses obtained are readily visualized in the percentage breakdown below (Figure A2). Continued forward movement at a constant rate (#2), a pause in movement (#17), and continued forward movement at a faster rate (#18) were observed most frequently; however, contact with the head frequently resulted in the larvae crawling backwards without turning around (#3) or turning sideways (#5). A qualitative assessment among the strains indicated that, compared to other strains, CHA>ORK1 larvae responded with the highest repeated response when touched on the anterior region (i.e., head), while both D42>ORK1 and Chord>ORK1 larvae, when tapped on the caudal end (i.e., tail), appeared to continue their forward motion at a faster rate.

For this assay, only the parental strain (UAS-ORK1) and the ORK-overexpressing crosses were examined, as this was intended only to determine whether this assay might be of use for further investigations and whether such a detailed ethogram of bodily movements was practical. Further investigation could utilize a wider range of genetic crosses, if desired.

### 3.4. Physiological Assay: Cardiac Rates

**Figure 5:**
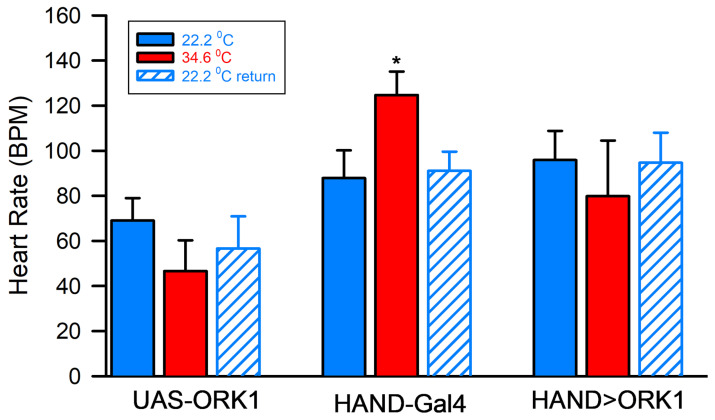
The effect of temperature on heart rate. HAND>ORK1 larvae had a significantly higher average heart rate than UAS-ORK1, but neither showed significant effects upon temperature alteration due to high variability among the preparations. HAND-GAL4 preparations, on the other hand, underwent a significant increase in heart rate given an increase in temperature (* *p* > 0.05, *T*-test, *n* = 10 for all strains).

The acute effects of increasing temperature on heart rate were observed for cardiac-specific ORK1 overexpression (HAND>ORK1) and compared with single-transgene outcross animals. On average, HAND>ORK1 organisms were observed to have a higher cardiac rate than UAS-ORK1 preparations, but changing the environmental temperatures from 22.2 °C to 34.6 °C and back did not produce any significant effects for either; on the other hand, HAND-GAL4 preparations underwent a significant increase in heart rate with the same temperature increase (Figure 5, * *p* > 0.05, *T*-test, *n* = 10 for all strains).

### 3.5. Video of Response to White Light

While dissecting larvae for the cardiac studies reported herein, it became clear that exposing the heart tube to bright white light via the microscope caused larvae of the M6-M7>ORK1 strain to contract, while UAS-ORK and BG487-GAL4 (i.e., M6-M7-GAL4) larvae did not respond similarly. This differentiation became more apparent after dark-adapting the larvae and (subsequently) shining bright white light on the crawling larvae. When conducting the body wall locomotion assay described above, a white light was used (though it was kept constant and minimal for all strains surveyed); it thus seems possible that the dim white light may have played a role in the reduced locomotive rates observed with the M6-M7>ORK1 strain. To better study the effects of the light used in dissection, a video was taken to allow for a comparison of the behaviors across the lines (i.e., crosses vs. parental).

In this clip (https://www.youtube.com/watch?v=1kPNNaoVSOo (accessed on 28 July 2025), M6-M7>ORK1 and UAS-ORK larvae are shown first individually and then together, first with infrared lighting and then with white light as well. Locomotive rates were not quantified within these groupings; rather, this was intended to demonstrate the effects of bright white light on the dark-adapted larvae. These behavioral differences made the dissection process slightly more complicated while exposing the heart tubes or body wall muscles. Hopefully, knowledge of this light sensitivity will benefit any future studies using this *Drosophila* strain.

### 3.6. Membrane Potential with Temperature

**Figure 6:**
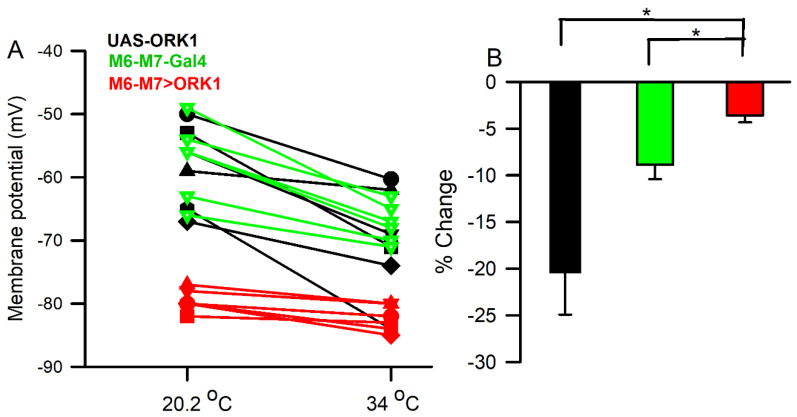
The changes in membrane potential observed with increased environmental temperature. (**A**) The membrane potential for individual UAS-ORK1, M6-M7 -GAL4 and M6-M7>ORK1 preparations when temperature is changed from 20.2 °C to 34 °C, shown as line graphs. (**B**) The percent changes for both strains, comparing initial values to those observed with increased temperature; this was greater for the parental strains (UAS-ORK1 and M6-M7-GAL4) compared to M6-M7>ORK1 (* *T*-test *p* < 0.05, *n* = 6).

In UAS-ORK1 and M6-M7-GAL4 larvae, resting membrane potential was observed to hyperpolarize when the bathing medium (20 °C) was replaced with a hotter one, at 33 °C (Figure 6). The M6-M7>ORK1 strain did not hyperpolarize as severely as the UAS-ORK1 parental strain or the M6-M7-GAL4 (Figure 6); however, given that this strain features a more negative resting membrane potential under normal conditions, this is not unexpected. Line graphs for individual larvae exhibit a significant change for both strains (*p* < 0.05, paired *T*-test, *n* = 6 for both groups), with a larger percent change observed in UAS-ORK1.

### 3.7. Developmental Studies

**Figure 7:**
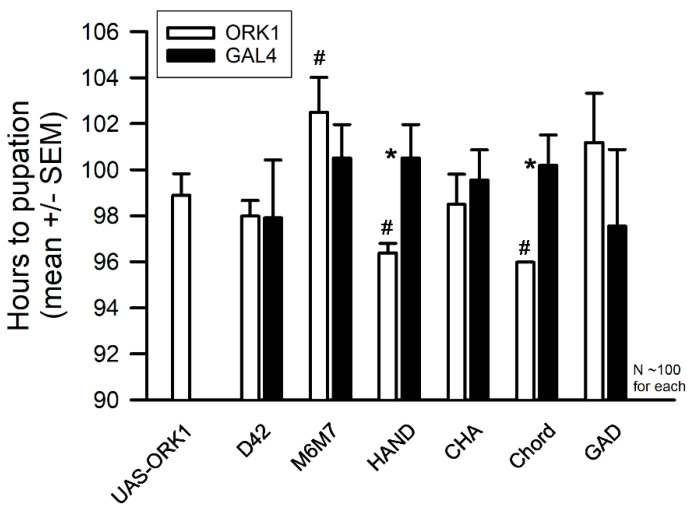
The developmental times from first instar to pupa for the various strains. Developmental time (hours) for the control single transgene outcross and K2p-overexpressing animals are shown. An enlargement of the *Y*-axis allows a better view of the variation among the strains. Ten vials (each containing ten first instars) were examined for each line, providing a total of around 100 larvae, and the average time for each vial was calculated for comparisons. There were no significant differences observed within the control outcross group (ANOVA, *p* = 0.952), while comparison within the ORK1 overexpressing lines indicated that only M6M7>ORK1, HAND>ORK1, and CORD>ORK1 were different (ANOVA, # *p* < 0.05). Between the ORK1 and GAL4 lines within a given tissue, only the HAND and Chord lines were significantly different (* *p* < 0.05, *T*-test).

The measure of time from first instar to pupa did not reveal large differences between the background strains and the ORK1 overexpressors (Figure 7). Within the control outcrosses, no statistical differences were observed, as determined using the Friedman Repeated Measures Analysis of Variance on ranks (*p* = 0.952) and the data was not normally distributed (Shapiro–Wilk). Between the ORK1 and GAL4 lines within a given tissue, only the HAND and Chord lines were significantly different (* *p* < 0.05, *T*-test). For ORK1 over-expression, on the other hand, M6M7>ORK1, HAND>ORK1 and Chord>ORK1 were significantly different (*p* = 0.031; ANOVA on ranks followed by Tukey’s Test for all comparisons). The UAS-ORK1 were compared to the ORK1 overexpressors, showing significant differences from M6M7>ORK1, HAND>ORK1, and Chord>ORK1 (Figure 7).

## 4. Discussion

This study demonstrated that *Drosophila* ORK1 overexpression in various tissues had variable effects on the behaviors and physiology of the larvae and adults studied. Using the GAL4-UAS system for altering gene expression, dORK1 K2P channels were overexpressed in muscle cells, motor and sensory neurons, and cardiac cells. Each of these crosses were subjected to a range of behavioral assays (to assess climbing rates, mouth hook movements, body wall movements, and response to touch) and physiological assessments (cardiac rate and membrane potential). Organism responses to temperature changes were also observed, as additional stress allowed for a better understanding of behavioral and physiological differences from the channel overexpression. It should be noted that this study served only as a preliminary survey; it was intended largely to provide insight into the general effects of altered channel expression as preparation for future studies into specific tissues and assays to use.

The ORK1 subtype found in *Drosophila* has not yet been characterized using mammalian categories because it currently lacks a complete pharmacological and physiological profile [16,17,55]. However, in previous studies, doxapram was shown to depolarize body wall muscles in larval *Drosophila* [33] and, with ORK1 overexpression in the muscle, produce larger depolarizations than those seen in the parental UAS-ORK1 lines [34]. This suggests that ORK1 K2P channels may behave similarly to the TASK K2P subtype found in mammalian body wall muscles. This finding is additionally corroborated by the ORK1 channel’s reaction to an acidic saline solution (pH~5), wherein the muscle membrane again depolarized upon exposure [56]. It should be noted that ORK1 is likely not the only K2P channel subtype expressed in *Drosophila* muscle membranes, as not even exposure to acidic doxapram (pH~5) depolarized the membrane potential to 0 mV or to the Na^+^ equilibrium potential. Other larval K2P subtypes may include TREK-1, as Fluoxetine (a known mammalian TREK-1 K2P channel blocker) was previously found to depolarize larval body wall muscles without blocking ORK1 channel activity [34,57,58].

The K2P subtypes expressed in larval *Drosophila*—be they ORK1 or one of the other yet-unidentified subtypes—are known to be activated by lipopolysaccharides (LPSs) produced by *Serratia marcescens* Gram-negative bacteria [59,60,61,62,63]. The precise expression levels of the eleven K2P channel subtypes in the *Drosophila* genome have not yet been precisely characterized across the various tissues [41,64,65] but each tissue likely expresses a mix. In the future, pharmacological and expression profiling in selective tissues could help characterize *Drosophila* K2P subtypes.

The climbing assays performed suggested that significant differences were obtained with CHA-GAL4 and D42>ORK1 larvae (compared to parental) as well as for D42- GAL4 vs. D42>ORK1, D42-GAL4 vs. m6m7-GAL4, ppk-GAL4 vs. CHA-GAL4, ppk-GAL4 vs. D42>ORK1, and CHA>ORK1 vs. CHA-GAL4. Temperature stress resulted in increased body wall movements in UAS-ORK1 and all GAL4 crosses except the CHA- GAL4 strain, while mouth hook movements increased in lines with ORK1 overexpression (except GAD>ORK1) and saw no change in most control outcrosses (except D42 and Chord, which increased). It is surprising to note that these results suggest differential effects of temperature alteration between locomotion rates and eating rates, as one would expect the motor neurons involved with eating to exhibit a similar response to those involved with the movement of body wall muscles. It is also interesting to note that the mouth hook movement rate was incredibly low for M6M7>ORK1 organisms as compared to UAS-ORK1 at both temperatures, and that the temperature-induced rate increase of 78% was also much larger than that observed in other cell types. Despite this, the cross only demonstrated reduced body wall movements when stressed at 33 °C. Also of note is the fact that the M6M7>ORK1 larvae were observed to respond uniquely when exposed to bright white light, as the larvae curled in on themselves and moved less frequently; this mannerism was not mimicked by any other strain in the study. ORK1-overexpressing lines likely did not increase locomotion with heat exposure due to hyperpolarization of the cells beyond that explainable by the increased density of ORK1 channels in the membrane, as shown with intracellular recordings made on muscle fibers.

The HAT behavioral assay suggests that the different crosses engaged in different behaviors under stressed conditions: UAS-ORK1 organisms tended to move forward or pause in response to touch, especially on the tail or abdomen, while D42>ORK1 organisms tended to favor turning or moving more quickly.

Physiological investigations also illustrated differences in the crosses overexpressing ORK1 channels. The HAND>ORK1 strain bore higher heart rates on average than those observed in the parental UAS-ORK1 strain, though neither group illustrated significant response to temperature alteration. It was also shown that the m6m7>ORK1 strain illustrated less hyperpolarization than UAS-ORK1 organisms when both were exposed to higher temperatures; this is likely because the former bears lower (i.e., more negative) resting membrane potential than the latter, even in normal physiological conditions. It should be noted that rapid temperature changes affect a cell’s membrane potential by influencing channel permeability, including changing the driving gradient of K^+^ in such a way as to render the E_K_ value more negative. In contrast, E_Na_ is driven to a more positive value by the same change, suggesting that NALCN (sodium leak channel) permeability changes as well. These alterations may, in turn, counteract some of the changes caused by E_K_ alterations.

The changes in membrane potential observed with altered temperature are likely not due to synaptic transmission, as the motor nerve itself is not being stimulated; indeed, it is likely that these effects stem from the innate biophysical properties of the muscle cell itself. However, the magnitude of the potential changes makes it difficult to investigate this possibility in an intact organism, for not only would temperature changes need to be recorded, but so, too, would the activity of the nervous system—and the ionic fluxes related to it—need to be measured continuously throughout [66]. Rapid temperature changes also induce conformational changes in the proteins for voltage-gated channels and their activation status [23]. While not every inter-membrane channel may exhibit such strong temperature-induced responses, K2P channels are known to have a gating relationship with temperature, and subtypes of these channels are known to feature varied reactions as well [24].

Important to note is the fact that, while the GAL4-UAS-driven gene expression used here does have some temperature dependence, the differences produced by the altered K2P channel expression occurred during the organism’s maturation (i.e., across the entire developmental period, which occurred at 21 °C). One would not expect this expression system’s slight temperature dependence to exhibit a substantial change in K2P channel expression and function during the brief (~1 min) period in which these behavioral assays were conducted. Additionally, all larvae were transferred individually from the 21 °C environment to one with warmer temperatures, so each experienced the same duration of temperature change during the assays. The variations in heart rate and behavior reported herein are, therefore, most likely independent from acute expression differences.

The effects of altered temperature on larval heart rate can be uniquely complicated, as many factors may be involved. This can be well demonstrated with consideration of the regulation behind Ca^2+^ dynamics and pacing potential. Pacing potential is highly dependent upon many channels functioning in concert, all of which must be functioning precisely, as well as the contractile machinery necessary for contractions to be observed at all; thus, is it visible that many components of the larval heart rate may be affected by temperature changes.

Even in this study, in which only temperatures of 21 °C and 33 °C were used to observe heart rates during ORK1 overexpression, a number of factors could be responsible for the effects reported. For example, it has previously been noted that cold-conditioned larvae (10 °C) undergoing acute exposure to a higher temperature (21 °C) present with a significant increase in heart rate [67]. Additionally, if the rate increases too quickly, the heart stops beating entirely, which is assumed to be caused by too large of an ionic imbalance [68,69]. In future, it may be worthwhile to test whether slowly and consistently raising the temperature would reveal a “breaking point” at which certain preparations deviate from the typical heart rate pattern.

Misexpression of certain K2P channels has been related to a number of pathologies in humans; the TASK-3 subtype, for example, has been linked with forms of cancer and some neurological disorders [13]. Additionally, other kinds of altered ion channel expression (such as the misexpression of NALCN sodium leak channels) has been connected to degenerative neurological conditions [41,42,43,44,45]. Further research into K2P channels (as well as ion channels more generally) thus provides insight into general physiology and that under altered biological conditions.

Knocking down or overexpressing genes involved with K2P channel formation within individual tissues allows for characterization of how altered channel expression might affect behavioral and physiological properties; in turn, this allows for predictions to be made on how pathological states (bearing altered expression) may affect tissues. However, it should be noted that the ORK1 strain used herein co-expresses GFP with the K2P channels, such that both the ORK1 protein and GFP are present in greater quantities than can be considered physiologically normal. It is possible that this could affect cellular functions in a manner for which this investigation could not account. GFP expression has been known to cause certain issues with cellular properties that, while known, are exceedingly hard to address [70]; higher protein expression may impede other proteins and influence membrane fluidity due to the insertion of additional proteins into the membrane. Since the temperature alterations implemented herein occur in addition to these other factors, some confounding issues may be present that should be addressed in future studies. Finally, since the K2P channel overexpression levels could not be precisely quantified, it would be of interest to expand this research with an investigation into how channel expression in the muscles coordinating mouth hook movements relates to those in the body wall muscles used for locomotion.

## Data Availability

The raw data supporting the conclusions of this article will be made available by the authors on request.

## Appendix A

The appendix contains graphical representations, and the coded number of the rubric used for indexing the larval behaviors.
